# Scratching increases epidermal neuronal branching and alters psychophysical testing responses in atopic dermatitis and brachioradial pruritus

**DOI:** 10.3389/fnmol.2023.1260345

**Published:** 2023-09-19

**Authors:** Lina Renkhold, Henning Wiegmann, Bettina Pfleiderer, Aysenur Süer, Claudia Zeidler, Manuel P. Pereira, Martin Schmelz, Sonja Ständer, Konstantin Agelopoulos

**Affiliations:** ^1^Department of Dermatology and Centre for Chronic Pruritus, University Hospital Münster, Münster, Germany; ^2^Clinic of Radiology, Medical Faculty, University Hospital Münster, University of Münster, Münster, Germany; ^3^Institute of Medical Informatics, University of Münster, Münster, Germany; ^4^Institute of Allergology, Charité – Universitätsmedizin Berlin, Corporate Member of Freie Universität Berlin and Humboldt-Universität zu Berlin, Berlin, Germany; ^5^Allergology and Immunology, Fraunhofer Institute for Translational Medicine and Pharmacology ITMP, Berlin, Germany; ^6^Department of Experimental Pain Research, MCTN, Faculty of Medicine Mannheim, University of Heidelberg, Mannheim, Germany

**Keywords:** chronic pruritus (CP), chronic scratching, lichenification, atopic dermatitis (AD), brachioradial pruritus, IENF density, IENF branching, dysesthesias

## Abstract

**Background:**

Chronic scratching imposes a major stress on the skin and can lead to itch intensity worsening, and consequently, patients may enter an itch–scratch cycle. This repetitive mechanical stress can result in lichenification, worsening of epidermal barrier function, and enhanced cutaneous inflammation. Furthermore, a reduction of intraepidermal nerve fibers was previously described in lichenification.

**Aim:**

The aim of this study was to investigate the influence of chronic scratching on the epidermal neuroanatomy and on sensory changes, in particular the prevalence of hyperknesis and alloknesis in patients after mechanical, chemical, and electrical stimuli.

**Methods:**

Analyses were performed on pruritic lichenified (chronically scratched), pruritic non-lichenified (not chronically scratched), and non-pruritic non-lesional (unaffected) skin areas of patients with inflammatory pruritus, i.e., atopic dermatitis (*n* = 35), and neuropathic pruritus, i.e., brachioradial pruritus (*n* = 34) vs. healthy matched controls (*n* = 64). Our fine-grained spatial skin characterization enabled specifically studying the differential effects of chronic scratching in inflammatory and neuropathic itch.

**Results:**

Analysis of intraepidermal nerve fiber density showed rarefaction of fibers in all three skin areas of patients compared with healthy controls in both diagnoses. Even more, the two pruritic areas had significantly less nerve fibers than the unaffected skin, whereas electrically induced itch was massively increased. Epidermal branching of the remaining nerve fibers in lichenified/chronically scratched skin was increased, particularly in patients with brachioradial pruritus, which may contribute to the pronounced local neuronal sensitivity. Hyperknesis and alloknesis were found to increase independently of lichenification.

**Conclusion:**

Our results indicate that chronic scratching may not affect intraepidermal nerve fiber density but leads to a stronger branching pattern of intraepidermal nerve fibers, which may contribute to local hypersensitivity. The increased sensitivity in the pruritic areas suggests mechanisms of peripheral sensitization, whereas the increased sensation of electrically and chemically induced itch in unaffected skin indicates central sensitization for itch.

## Introduction

Scratching is a physiologic response to relieve the unpleasant itch sensation (bin Saif et al., 2012). However, persistent and excessive scratching can further worsen the itch by promoting the release of pro-inflammatory effectors (Feng et al., 2022), thus, resulting in an itch–scratch cycle that can lead to an impairment of the skin barrier and lichenification, characterized by the promotion of inflammation, epidermal hyperplasia, and neuropathy (Yosipovitch and Bernhard, 2013; Mack and Kim, 2018; Rinaldi, 2019). Accordingly, it is assumed that scratching directly influences neuroanatomical characteristics in pruritic skin. Previously, we could demonstrate a profound rarefication of intraepidermal nerve fibers (IENFs) in the pruritic skin of patients with atopic dermatitis (AD) as well as brachioradial pruritus (BRP) (Pogatzki-Zahn et al., 2020; Agelopoulos et al., 2022). Remarkably, not only the intraepidermal nerve fiber density (IENFD) was altered, but also the branching of the remaining epidermal nerve fibers was found increased in pruritic skin, particularly in BRP patients (Agelopoulos et al., 2022; Ronchi et al., 2023). Altered neuroanatomy is not only discussed as playing a role in the pathogenesis of itch but is also linked to sensitization-related dysesthesias, i.e., alloknesis and hyperknesis. Both of these dysesthesias can frequently be observed in chronic pruritus patients (Ikoma et al., 2006; Andersen et al., 2018). Alloknesis is defined as the perception of itch induced by innocuous, non-itchy stimulation (LaMotte, 1992; Andersen et al., 2017b). It is assumed that itch transmission occurs via spinal processing of low-threshold Aβ-fiber input close to symptomatic skin. Hyperknesis, another dysesthesia, is characterized by an increased itch sensation after an itchy stimulus that is thought to predominantly activate sensitized Aδ- and C-fibers (Andersen et al., 2018). The test stimuli for hyperknesis or alloknesis can be of different origins, e.g., chemical (Andersen et al., 2017b), mechanical (Fukuoka et al., 2013; Pall et al., 2015), or electrical (Andersen et al., 2018; Solinski and Rukwied, 2021).

To assess in greater detail the impact of chronic scratching in the pathogenesis and chronicity of pruritus, we investigated the neuroanatomy and the perceived intensities of dysesthesias separately in those skin areas that were either pruritic and lichenified (PLi) as a consequence of chronic scratching, pruritic, but non-lichenified (PNLi), or non-pruritic non-lesional (NPNL) areas. We included patients with chronic inflammatory pruritus, i.e., AD or neuropathic pruritus, i.e., BRP to investigate the influence of scratching on patients with different underlying diseases.

## Materials and methods

### Study population

Adult patients with CP, AD, or BRP, as well as sex- and age-matched healthy controls (HCs), were enrolled in this study. Patients were recruited at the Center for Chronic Pruritus, Department of Dermatology, University Hospital Münster (Germany), and HC via advertisements. A detailed explanation of inclusion and exclusion criteria is shown in Supplementary Table 1. The study was approved by the local Ethics Committee (Medical Faculty of the University of Münster; No: 2017-562-f-S) and registered at the German Clinical Trials Register (DRKS; No: DRKS00014745). Patients signed an informed consent form prior to the start of the study. All study procedures were performed according to the Declaration of Helsinki and its later revisions.

### Study design

In this prospective clinical study, after verification of the inclusion and exclusion criteria, a board-certified dermatologist took the medical history and performed a comprehensive physical and dermatological examination. Assessment areas on the arms were then determined for patients and HCs. Experimental procedures followed a fixed, predetermined order. First, a chemical skin challenge with cowhage was performed in non-pruritic non-lesional skin (NPNL) of all study participants. In AD and BRP patients, all other assessments were performed on pruritic lichenified skin (PLi), pruritic non-lichenified skin (PNLi), and NPNL skin. Assessments in HCs were done in the NPNL area at the same anatomical location as the PLi area of the matched patient.

We assessed electrical hyperknesis by stimulating with half-sine and sine wave pulses. Subsequently, alloknesis was tested by stimulating the skin with a cotton swab in all assessment areas, and we used Von Frey filaments to determine the mechanical pruritus/pain threshold in NPNL. Finally, biopsies were taken from all assessment areas in patients and controls to analyze neurocutaneous anatomy.

### Experimental procedures

#### Clinical parameters

The average and worst pruritus intensity of the previous 24 h was assessed with the numerical rating scale (NRS; 0–10) and the categorical verbal rating scale (VRS; 0: no itch, 1: weak, 2: moderate, 3: severe, and 4: very severe). Additionally, the duration of pruritus in months was recorded. We evaluated the overall severity of scratch lesions using the Scratch Sign Score (SSS; 0–20), in which the percentage of the affected body surface and the morphology of scratch lesions were assessed (Supplementary Table 2).

#### Stimulation with cowhage

Patients and HC were stimulated with cowhage at the NPNL skin of the ventral forearm in order to detect chemical hyperknesis. Approximately 5–10 cowhage spicules fixed on a cotton swab were applied to the skin. Patients were instructed to report the induced pruritus intensity on the NRS (0–10) every minute for 10 min. The area under the curve (AUC) and the maximal itch were used for analyses.

#### Mechanical alloknesis

To assess alloknesis, we gently stroked the skin with a cotton swab at all testing sites and asked the patients whether pruritus was perceived. If so, the intensity of pruritus was assessed using a NRS scale (0–10). We performed three assessments in each area. Average and maximal intensities were considered for analysis.

#### Mechanical pruritic/pain threshold

The mechanical pruritic/pain threshold was assessed at NPNL in patients and HCs using von Frey filaments (force: 0.008 g to 300 g; Semmes-Weinstein Monofilaments/Touch-Test Sensory Evaluators, North Coast Medical Inc., Morgan Hill, CA, USA) according to the “up and down method” (Dixon, 1980). Filaments were applied to the skin for 1 s at a 90° angle in ascending order until the patient reported pruritus or pain. Afterward, stimulation occurred in descending order of force until no pruritus or pain was perceived. The geometric mean of five supra-threshold and five sub-threshold scores was calculated in order to determine the mechanical pruritic/pain threshold.

#### Electrical stimulation

The electrical stimulation protocol was adapted from previous studies (Rukwied et al., 2020). In brief, electrical stimulation was performed using a constant current stimulator (Digitimer DS5, Welwyn Garden City, UK) connected to a Digital-Analogue Converter (DAQ NI USB-6221, National Instruments, Austin, TX, USA) controlled by Dapsys 8 software (www.dapsys.net). We started with an instruction and training session familiarizing study participants with the procedure. We documented the pruritus or pain intensities on a NRS (0–10) after each electrical stimulus, respectively. The patients were instructed to separately report the sensation of pruritus or pain and its respective intensity.

Single half-sine wave pulses (500 ms, 0.2–0.4–0.6–0.8–1 mA) were applied twice on all testing areas using a transcutaneous electrode (pair of rounded bipolar platinum electrodes, diameter 0.4 mm, distance 2 mm, Nørresundby, Denmark). The evoked average maximal itch or pain and the slope of the symptom intensity with increasing electric current were calculated.

Study participants received sine wave pulses (10 pulses, 4 Hz, 0.005–0.01–0.025–0.05–0.1–0.2–0.4 mA) in order to detect the sine perception threshold. When a sensation was perceived, study participants were asked to qualify it as an itch, pain, burning, stinging, or pulsing. Afterward, sine wave pulses (10 pulses, 4 Hz, 0.025–0.05–0.1–0.2–0.4 mA) were applied, and the evoked maximal itch or pain intensity was recorded using the NRS.

#### Determination of the intraepidermal nerve fiber density

Intraepidermal nerve fiber density (IENFD) is calculated as the number of nerve fibers crossing the basement membrane from the dermis to the epidermis in relation to the length of the epidermis. IENFD was determined as previously described (Schuhknecht et al., 2011; Bobko et al., 2016; Pogatzki-Zahn et al., 2020). Briefly, skin was obtained from all testing sites of patients and HC via 6 mm punch biopsies. One-third of the biopsy was used within this study for neuroanatomical analyses, while the rest of the sample was analyzed in other experiments (data not shown). Skin samples were fixed in paraformaldehyde overnight, buffered in sucrose, and snap-frozen in liquid nitrogen. Each of the three 30 μm cryosections per biopsy was incubated with a primary antibody targeting protein gene product (PGP) 9.5 (polyclonal rabbit, 1:2,000; Chemicon, Temecula, CA, USA). The sections were stained with a second antibody, fluorescein isothiocyanate isomer 1 (FITC)-conjugated Swine Anti-Rabbit (1:200, Dako, Cytomation, Glostrup, Denmark). Intraepidermal nerve fibers from three sections were counted at 400x magnification using a fluorescence microscope (Olympus, Modell BX43F, Tokyo, Japan). The length of the epidermis was measured with the software cellSens Dimension at 200x magnification using the same microscope. To calculate the IENFD, mean counts were divided by mean epidermal length.

#### Neuronal branching

Using the stained cryosections mentioned above, branching patterns were assessed semi-quantitatively considering epidermal nerve length and sprouting at 400x magnification (Olympus, Modell BX43F, Tokyo, Japan). We categorized branching patterns into four groups according to the predominant pattern, as follows: linear only, mainly linear, mainly branched, and only branched.

### Statistics

Statistical analyses were performed with IBM SPSS Statistics for Windows, version 27.0 (Armonk, NY, USA). We performed group comparisons between patient groups with the Mann–Whitney *U*-test, while for comparisons between dependent samples, the Wilcoxon signed-rank test was used. We used the chi-square test, as appropriate, for comparison of categorical variables between groups. Data are shown as median [interquartile range (IQR)]. Correlations were calculated by Spearman rank correlation. Statistical significance was set at a *p*-value of < 0.05.

## Results

### Study population

We included 69 patients (AD = 35; BRP = 34) and 60 age- and sex-matched healthy volunteers. Patients with BRP were significantly older than patients with AD (*p* = 0.002) but showed a shorter duration of pruritus (*p* < 0.001). There were no differences in pruritus intensity or in the scratch sign score (SSS) between AD and BRP patients. Demographic data and pruritus characteristics are summarized in Table 1.

**Table 1:** Demographic data and pruritus characteristics.

**Data**		**AD**	**AD-matched controls**	**BRP**	**BRP-matched controls**
N		35	31	34	33
Sex	m:f	23:12	21:10	11:23	11:22
Age	Years	43.0^**^ [27.0;59.0]	42.0 [25;57]	61.5^**^ [53.3;67.0]	61 [52.0;64.0]
Pruritus duration	Months	259.5^***^ [67.0;468.3]	NA	47.5^***^ [21.0;125.8]	NA
Pruritus intensity (24 h)	VRS_mean_	2.0 [2.0;3.0]	NA	2.0 [2.0;3.0]	NA
	VRS_max_	3.0 [2.0;3.0]	NA	2.0 [2.0;3.0]	NA
	NRS_mean_	7.0 [4.8;8.0]	NA	6.0 [3.5;8.0]	NA
	NRS_max_	8.0 [5.0;8.0]	NA	7.0 [5.0;8.5]	NA
Scratch sign score	SSS	6.0 [4.0;12.0]	NA	8.0 [4.0;11.0]	NA

Data are shown as median [IQR] for AD and BRP patients and sex- and age-matched healthy controls. AD, atopic dermatitis; BRP, brachioradial pruritus; f, female; IQR, interquartile range; m, male; NA, not applicable; NRS, numerical rating scale [range, 0–10]; SSS, scratch sign score [range, 0–20]; VRS, verbal rating scale [0 = no itch, 1 = low itch, 2 = moderate itch, 3 = severe itch, and 4 = very severe itch]. Differences between AD and BRP patients marked in bold, Mann–Whitney *U-*test, ^**^p < 0.01, ^***^p < 0.001.

### Neuronal architecture

**Figure 1:**
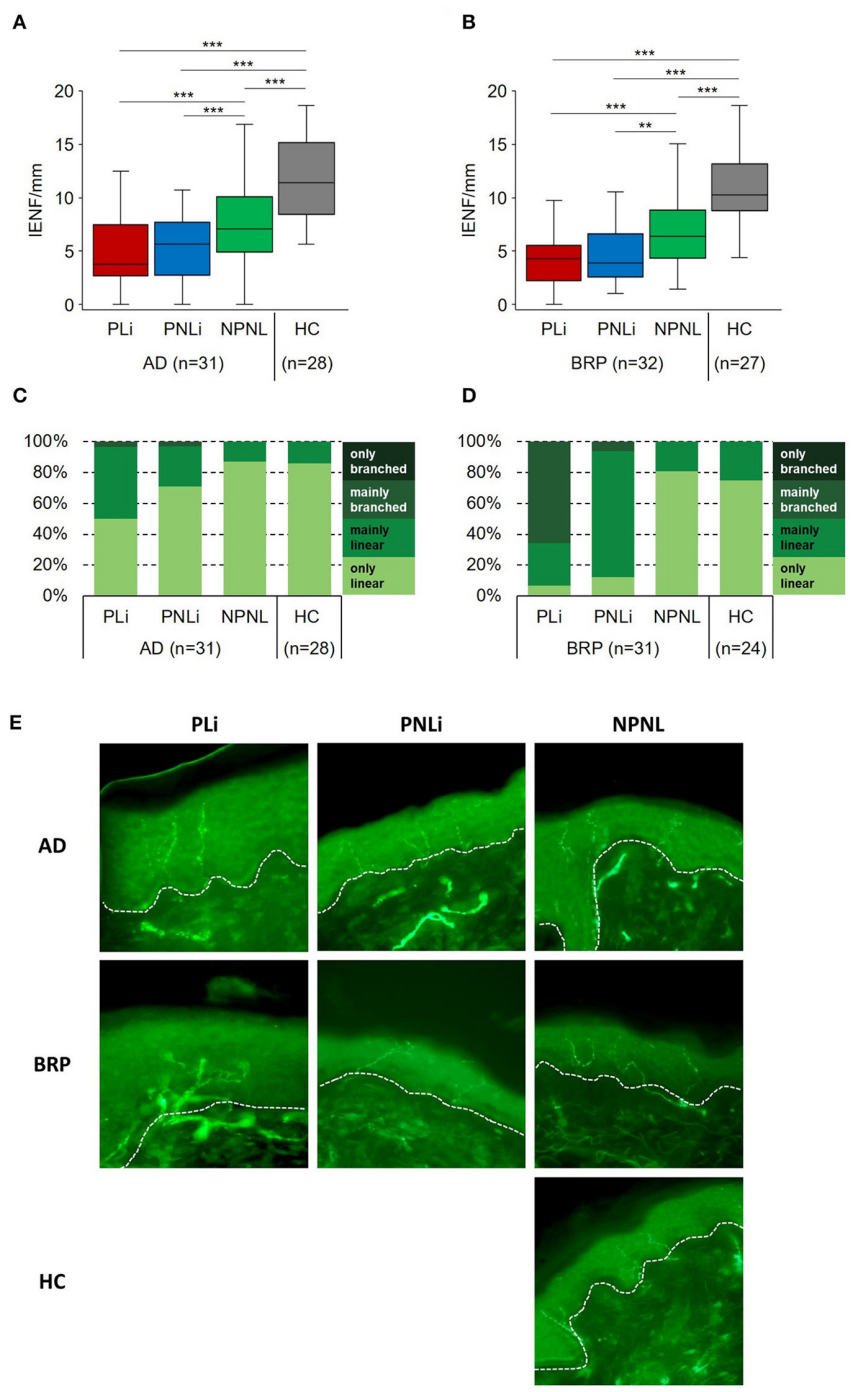
Fine-scale epidermal neuroanatomy of AD and BRP patients. **(A, B)** Boxplots of IENFD (AD: *n* = 31; BRP: *n* = 32) assessed at pruritic lichenified skin (PLi), pruritic non-lichenified skin (PNLi), and non-pruritic non-lesional skin (NPNL) from AD and BRP patients and in healthy skin of matched HCs (AD: *n* = 28, BRP: *n* = 27) are shown. **(C, D)** Stacked bar plots representing the proportion of branching categories of PLi, PNLi, and NPNL skin in AD (*n* = 31) and BRP (*n* = 31) patients and in healthy skin of matched HCs (AD: *n* = 28, BRP: *n* = 24) are presented. Pruritic skin of AD and BRP patients showed significantly reduced IENFD compared to healthy skin (*p* < 0.001). **(A**, **B)** In all skin areas, the IENFD of AD and BRP patients was significantly reduced compared to HCs (*p* < 0.001). **(C)** In AD patients, branching of intraepidermal fibers in PLi skin was more prominent than in PNLi (*p* = 0.002), NPNL (*p* = 0.001), and HCs (*p* = 0.004). **(D)** In BRP patients, branching of intraepidermal fibers in both PLi and PNLi skin was more abundant compared to NPNL (*p* < 0.001) and HCs (*p* < 0.001). **(E)** Representative immunofluorescent stainings of IENF (PGP9.5 stained, green) show abundant branching of nerves in the PLi skin of patients, especially in BRP. In both patient groups, nerve fibers are less branched in PNLi skin, whereas nerve fibers are primarily linear in NPNL skin. Magnification: original x400, dashed lines = basement membrane. AD, atopic dermatitis; BRP, brachioradial pruritus; HC, healthy control; IENF, intraepidermal nerve fiber; IENFD, intraepidermal nerve fiber density; NPNL, non-pruritic non-lesional skin; PLi, pruritic lichenified skin; PNLi, pruritic non-lichenified skin. Related samples: Wilcoxon signed-rank test; independent samples: Mann–Whitney *U*-test. **p < 0.01, ***p < 0.001.

For the analysis of IENFD and epidermal branching, skin biopsies were analyzed from a total of 63 patients (AD: *n* = 31; BRP: *n* = 32) and from 28 AD-matched and 27 BRP-matched HCs (median [IQR] data are shown in Supplementary Table S3). In neither patient group differences between PLi and PNLi skin were observed, but both pruritic skin areas showed a significant reduction in IENFD compared to their NPNL skin (AD PLi/PNLi: *p* < 0.001; BRP PLi *p* < 0.001 and PNLi *p* = 0.002). Compared to matched HCs, the IENFD was significantly reduced in all three analyzed areas of the patients (AD: PLi/PNLi/NPNL *p* < 0.001; BRP: PLi/PNLi/NPNL *p* < 0.001; Figures 1A, B). Of note, a negative correlation between IENFD in PNLi skin and average pruritus intensity of the last 24 h in both AD (r = −0.558, *p* = 0.001) and BRP (*r* = –0.619, *p* < 0.001) patients was observed.

Additionally, the nerve fibers of patients were categorized based on their proportion of branched fibers within the epidermis (Figures 1C–E). The lichenified skin of BRP patients was most striking, as mainly branched nerve fibers were found in more than half of the samples. In these patients, branching was significantly more prominent in PLi as compared to PNLi skin (BRP: *p* < 0.001). The same difference was observed in the pruritic skin of AD patients (AD: PLi vs. PNLi *p* = 0.002). In NPNL skin, more than 80% of AD and BRP patients as well as HCs had mainly linear epidermal nerve fibers differing significantly from the pruritic (AD: PNLi vs. NPNL *p* = 0.034; BRP: PNLi vs. NPNL and HC *p* < 0.001) and scratched skin areas (AD: PLi vs. NPNL *p* = 0.001, vs. HC *p* = 0.004; BRP: PLi vs. NPNL and HC *p* < 0.001). Moreover, BRP patients had significantly more mainly linear nerve fibers in PNLi skin and more mainly branched fibers in PLi skin compared to the respective pruritic areas of AD patients (PLi and PNLi: *p* < 0.001).

### Chemical hyperknesis by stimulation with cowhage

**Figure 2:**
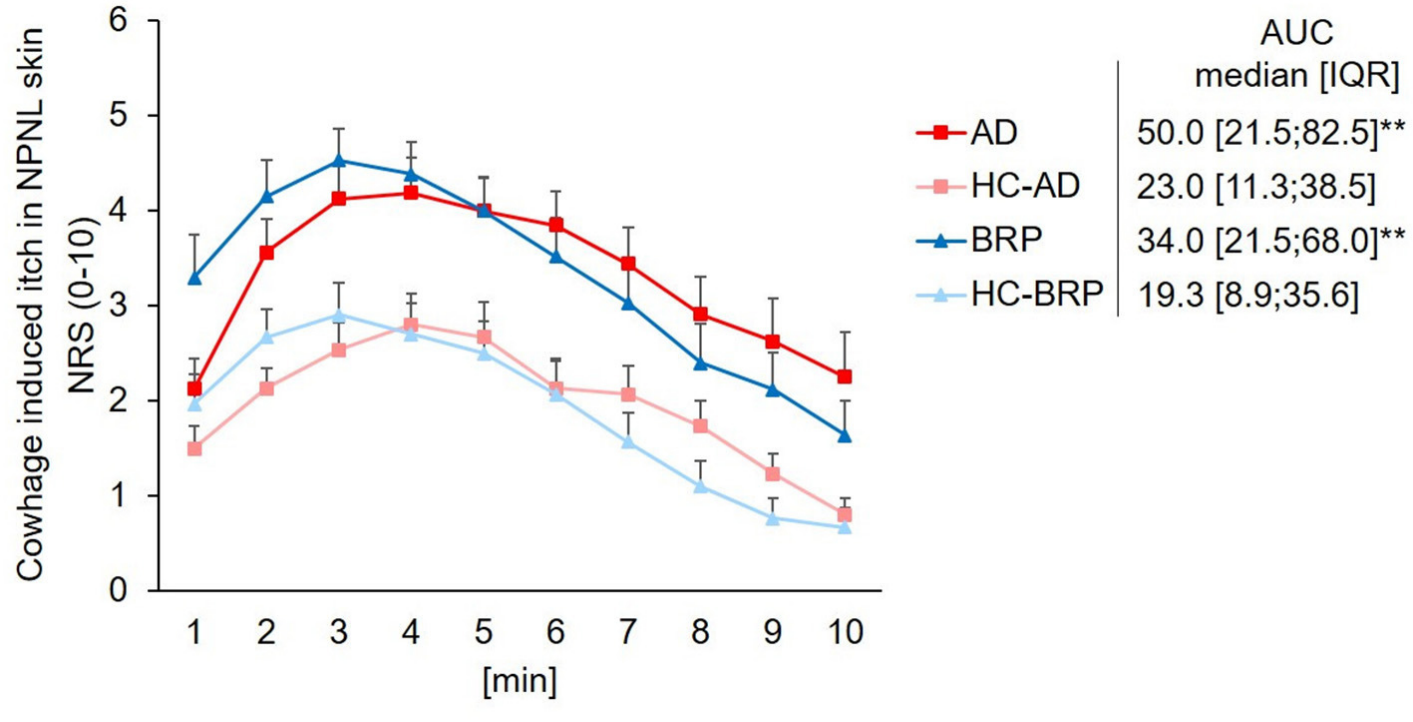
Chemical hyperknesis after stimulation with cowhage. Mean pruritus intensity (SEM) on the NRS (0–10) evoked by stimulation with cowhage over 10 min. Assessments were performed on NPNL skin at the ventral forearm of patients with AD (*n* = 34), BRP (*n* = 35), and matched HCs (AD-HCs *n* = 31 and BRP-HCs *n* = 33). Pruritus intensity was higher in AD (*p* = 0.003) and BRP (*p* = 0.002) patients compared to HCs, as assessed by the AUC. AD, atopic dermatitis; AUC, area under the curve; BRP, brachioradial pruritus; HCs, healthy controls; IQR, interquartile range; NPNL, non-pruritic non-lesional skin; NRS, numeric rating scale (range, 0–10); SEM, standard error of mean. Independent samples: Mann–Whitney U-test. **p < 0.01.

Stimulation with cowhage in the NPNL skin of patients (AD: *n* = 31; BRP: *n* = 33) led to higher itch intensities compared to stimulation at healthy skin of their matched HCs (HC-AD: *n* = 30 and HC-BRP: *n* = 31), as measured by the area under the curve (AUC; AD: *p* = 0.003; BRP: *p* = 0.002) and by the maximal perceived itch (AD and BRP: *p* < 0.001; Figure 2).

### Mechanical stimulation

#### Mechanical perception threshold of pruritus or pain (von Frey filaments)

We recorded no differences in mechanical pruritus/pain threshold assessed in NPNL skin of patients with AD and BRP compared to matched controls (AD: median target force 4.8 g [2.2; 28.5], *n* = 24 vs. HCs: median target force 38.2 g [4.4; 105.0], *n* = 22, *p* = 0.121; BRP: median target force 6.5 g [1.4; 33.8], *n* = 15 vs. HCs: median target force 6.8 g [1.4;97.0], *n* = 15, *p* = 0.885). Additionally, the thresholds of AD and BRP patients did not differ (*p* = 0.665).

#### Mechanical induced alloknesis (cotton swab)

**Figure 3:**
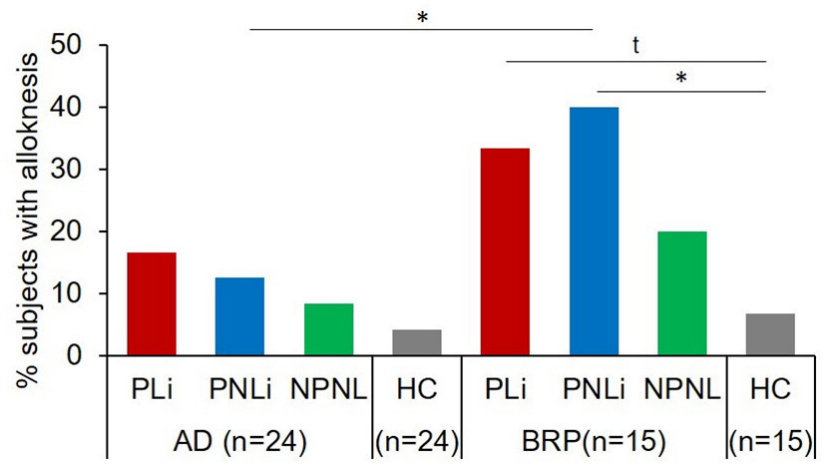
Mechanical alloknesis induced by stimulation with a cotton swab. Percentage of patients showing alloknesis after stimulation with a cotton swab at pruritic lichenified skin (PLi), pruritic non-lichenified skin (PNLi), and non-pruritic non-lesional skin (NPNL) in patients with AD (n = 24) and BRP (n = 15); and in healthy skin of matched controls (AD: n = 24, BRP: n = 15). AD patients showed no differences in the percentage of alloknesis across skin areas or compared to HCs. Alloknesis was more frequently recorded in pruritic areas of BRP patients compared to HCs (PLi vs. HC: p = 0.051; PNLi vs. HCs: p = 0.025). AD, atopic dermatitis; BRP, brachioradial pruritus; HCs, healthy controls; NPNL, non-pruritic non-lesional skin; PLi, pruritic lichenified skin; PNLi, pruritic non-lichenified skin. chi-square test, t, Trend p < 0.1; *p < 0.05.

The prevalence of alloknesis, defined as the percentage of patients perceiving itch after stimulation with a cotton swap in one of the three assessments, was higher in lichenified skin (33.33%; PLi vs. HC: *p* = 0.051) and significantly higher in pruritic, non-lichenified skin (40%) of BRP patients compared to HCs (6.66%; PNLi vs. HC: *p* = 0.025; Figure 3). No differences were observed across testing areas in AD patients or between AD and HCs. Significantly less AD patients perceived alloknesis in PNLi skin (12.5%) compared to BRP patients (40%, *p* = 0.047). In addition to the higher prevalence of alloknesis in the pruritic skin of BRP patients, the intensity of the mechanically induced itch was higher in their PLi as compared to NPNL skin (*p* = 0.038), but also when PNLi skin was compared to AD patients (*p* = 0.042) with maximal NRS ratings up to 5 in PLi, up to 4 in PNLi, and 2 in NPNL skin of BRP patients (Supplementary Figure S1). AD itch responders showed maximal NRS scores up to 3 in PNLi and 2 in PLi and NPNL skin.

### Electrical induced hyperknesis (transcutaneous half-sine and sine waves)

**Figure 4:**
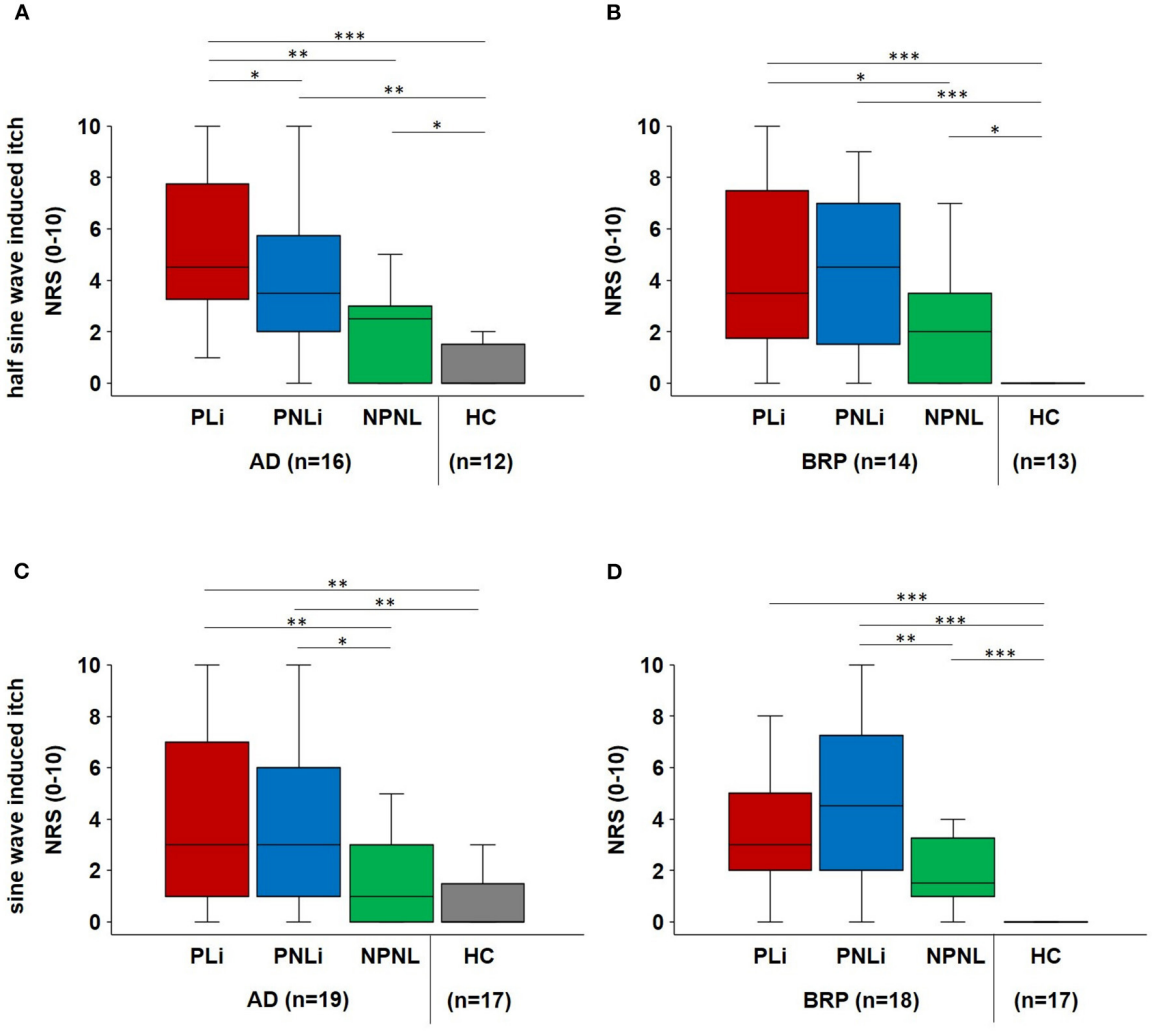
Electrical-induced hyperknesis. **(A**, **B)** Boxplots of electrical half-sine wave-induced maximal itch intensities (NRS) in PLi, PNLi, and NPNL skin of itch responders suffering from AD (n = 16) and BRP (n = 14), as well as in NPNL skin of matched HC (n = 12 and 13, respectively). **(C, D)** Electrical sine wave-induced maximal itch intensities (NRS) in PLi, PNLi, and NPNL skin of itch responders of AD (n = 19) and BRP (n = 18) as well as in NPNL skin of matched HC (n = 17 each). **(A)** Half-sine stimulation evoked increased itch in lichenified skin of AD patients compared to PNLi (p = 0.016), NPNL (p = 0.003), and controls (p < 0.001) as well as decreased itch intensities in controls as in PNLi (p = 0.003) and NPNL skin (p = 0.049). **(B)** In BRP patients, itch intensity was higher in lichenified skin compared to NPNL skin (p = 0.048) and HC (p < 0.001) and higher in both PNLi and NPNL skin compared to HCs (PNLi: p < 0.001; NPNL: p = 0.016). **(C)** According to sine wave stimulation of AD patients, higher NRS ratings were observed in lichenified skin as in NPNL skin (p = 0.002) and in HCs (p < 0.001) as well as in PNLi compared to NPNL skin (p = 0.032) and HCs (p = 0.004). **(D)** BRP-matched HCs had significantly lower itch ratings compared to BRP patients in all assessment areas (p < 0.001) and patients reported only increased itch intensity in PNLi skin compared to NPNL skin (p = 0.002). Itch responders were patients who reported at least one electrically induced itch during the stimulation paradigms. AD, atopic dermatitis; BRP, brachioradial pruritus; HC, healthy control; NPNL, non-pruritic non-lesional skin; NRS, numerical rating scale; PLi, pruritic lichenified skin; PNLi, pruritic non-lichenified skin. Related samples: Wilcoxon signed-rank test; independent samples: Mann–Whitney U-test. *p < 0.05, **p < 0.01, and ***p < 0.001.

The electrical stimulation paradigms, including half*-*sine and sine wave stimulation, were applied transcutaneously with increasing intensity, and the maximal evoked itch was assessed in all testing areas. Only the data of participants who responded at least once with itch were included in Figure 4 to highlight the skin condition-specific differences. Data for the whole cohort are shown in Supplementary Figure S2.

Upon half-sine stimulation that excites polymodal C-nociceptors, the maximal evoked itch was higher in PLi skin of AD patients compared to PNLi (*p* = 0.016) as well as NPNL (*p* = 0.003) and HC (*p* < 0.001; Figure 4A). In BRP patients, itch intensities did not differ between PLi and PNLi skin, but itch scores were higher in PLi skin compared to NPNL (*p* = 0.048) and in all skin areas compared to matched controls (PLi/PNLi: *p* < 0.001, NPNL: *p* = 0.016; Figure 4B). The maximal half*-*sine-evoked pain was higher in NPNL skin in BRP patients compared to controls (*p* = 0.025). Additionally, AD patients perceived significantly less pain in PLi (*p* = 0.044) and NPNL (*p* = 0.001) skin than BRP patients.

As for sine wave stimulation that excites polymodal and mechano-insensitive C-nociceptors, perception thresholds were significantly higher in the PLi skin of AD patients compared to their NPNL skin (*p* = 0.014).

Maximal sine wave-evoked itch did not differ significantly between PLi and PNLi skin in both AD and BRP patients. Itch scores were higher in PLi (*p* = 0.002) compared to NPNL in AD, and in both patient groups, we observed higher itch intensity in PNLi than in NPNL skin (AD: *p* = 0.032; BRP: *p* = 0.002; Figures 4C, D). In BRP patients, electrically evoked maximal pain was higher in PNLi compared to PLi skin (*p* = 0.021). Furthermore, sine wave-induced pain was increased at PNLi (*p* = 0.027) and NPNL (*p* = 0.03) in BRP compared to the corresponding sites in AD patients. Electrical-evoked pain data (median [IQR]) are shown in Supplementary Table S4.

**Figure 5:**
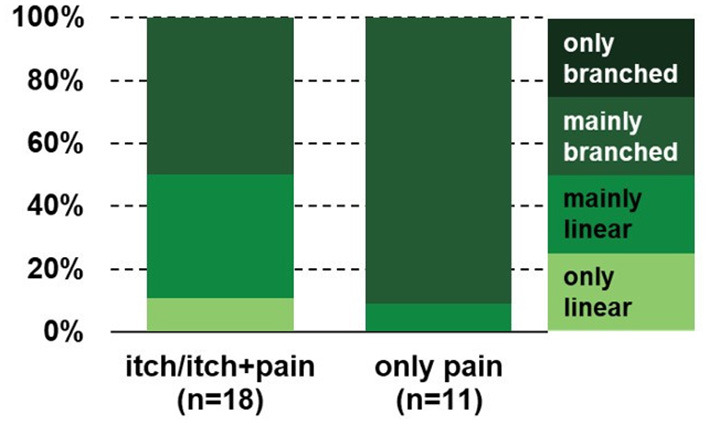
Branching pattern in the PLi skin of BRP patients reporting only pain or itch and pain after electrical stimulation. The branching pattern of patients who only perceive pain is significantly higher compared to those reporting itch alone or both itch and pain (p < 0.05, chi-square test). BRP, brachioradial pruritus; PLi, pruritic lichenified skin.

Remarkably, in the lichenified skin of BRP patients, nerve fibers were mainly branched in more than 80% of those patients reporting only pain after electrical stimulation, whereas in approximately 50% of patients perceiving only itch or itch and pain branching was mainly and only linear (*p* < 0.05; Figure 5), indicating that extreme branching of nociceptive endings increases their responses to electrical stimulation to such a degree that it is mainly felt as pain.

## Discussion

To date, the role of chronic scratching in the pathogenesis and chronicity of pruritus is not fully understood. To shed further light on this issue, we investigated the neuroanatomy and performed psychophysical measurements in the skin of patients with atopic dermatitis (AD) and brachioradial pruritus (BRP). We compared skin that was pruritic and chronically scratched and thus lichenified (pruritic lichenified = PLi) with pruritic but not chronically scratched (pruritic non-lichenified = PNLi) and with the unaffected skin of the patients (non-pruritic non-lesional = NPNL).

In previous studies, we and others have shown that both patients with AD and BRP have reduced intraepidermal nerve fiber density (IENFD) compared with healthy subjects (Tan et al., 2019; Pogatzki-Zahn et al., 2020). In this study, IENFD is defined by the number of nerve fibers crossing the basement membrane per mm epidermis. These results are also corroborated in this study. Our study also revealed that when assessing pruritus and its impact on patients, it is crucial to take into account the characteristics of individual skin areas. Pruritic skin (PLi and PNLi) had significantly lower IENFD compared to NPNL and HCs, but lichenification had no further impact on IENFD. Furthermore, in the NPNL skin of AD and BRP patients, IENFD was also significantly reduced as compared to HCs. It should be noted, that we also found a negative correlation between IENFD in PNLi skin and the average itch intensity of the last 24 h in both entities. This could be an indicator of a partial neuropathic component in AD, analogous to BRP (Pereira et al., 2020, 2021), and requires further investigation. Our finding may therefore suggest that the mechanical stress of chronic scratching does not further reduce IENFD. Rather, reduced IENF may be linked to neuropathy in BRP, inflammatory processes in AD, and the dysregulation of nerve plasticity-associated genes, as reflected in altered expression signatures that have already been described for AD and are also conceivable for BRP (Möbus et al., 2021; Agelopoulos et al., 2022). In contrast to IENFD, however, intraepidermal nerve fiber branching was increased in lichenified pruritic skin. This was particularly evident in BRP. An increased branching of injured nerve fibers has already been described as part of a regenerative process (Ronchi et al., 2023). Therefore, it is conceivable that nerve fibers in PLi skin injured by scratching would also respond with increased branching as a result of regeneration. However, such branching will decrease over time (Ronchi et al., 2023), which could also explain the reduced branching in PNLi skin (compared to PLi) that has not been chronically scratched. In AD, the branching pattern differentiated the different skin areas distinctly less. However, a greater number of patients revealed increased branching in PLi (vs. PNLi, NPNL, and HCs). The observation that patients with AD in this study showed mainly linear to only linear nerve fibers could be due to the presence of inflammation. Thus, it has already been described that specific secreted factors can reduce nerve fiber density in inflamed tissue (Fassold et al., 2009; Pongratz and Straub, 2014) and could also suppress branching. This is also confirmed by one of our previous studies, indicating that the gene *SEMA3A*, responsible for the collapse of nerve fibers, was significantly more expressed in the affected skin of patients with AD than in patients with BRP. Furthermore, *NGF*, the antagonist of *SEMA3A*, was upregulated in affected skin from BRP patients, which would be indicative of the increased branching pattern (Agelopoulos et al., 2022). Further studies are urgently needed here for a deeper understanding of the role of nerve fiber architecture in chronic pruritus and chronic scratching. To investigate whether the differences in neuroanatomy are also reflected in the function of the fibers, we performed psychophysical measurements.

Regarding cowhage stimulation (in NPNL only), both groups of patients showed a higher onset value (y-intercept) than HCs, and a BRP had the highest value. In BRP, the slope dropped analogously to HCs, but remained at a higher endpoint after 10 min. In contrast, the more rapid increase in AD patients may argue for a general non-histaminergic hypersensitivity in AD (Andersen et al., 2017a). Similarly, AD patients reported more intense pruritus over a longer period of time and decreased more slowly than BRP and HCs with a higher endpoint. This specific response in AD patients suggests that the underlying inflammation already causes subthreshold sensitization of polymodal C- and A-delta fibers even in the apparently unaffected area (Schmelz, 2013). In lesional skin of AD vs. healthy skin, an increased pruritus sensation with a long-lasting plateau phase was also observed in another study, which corroborates our findings (Andersen et al., 2017b). The increased sensation in BRP also argues for an already underlying increased sensitivity to cowhage, which is partly consistent with four previous studies, which however took place in the pruritic area (Pogatzki-Zahn et al., 2020). Furthermore, Rukwied et al. (2013) already demonstrated that NGF leads to an increased response to cowhage. Since an overexpression of NGF in BRP patients was shown (Agelopoulos et al., 2022), this could be a plausible explanation for the higher entry level in the NPNL area of BRP patients after chemical pruritus induction in our case. Alternatively, chronic pruritic input from the symptomatic skin may have induced central sensitization for itching.

AD patients reported alloknesis more frequently in the PLi area than in the PNLi and NPNL, and compared to HCs, the level did not reach statistical significance. In contrast, BRP patients reported alloknesis significantly more frequently in both pruritic areas as compared to NPNL. This was also reflected in higher perceived pruritus intensities in PLi. This finding is in line with the expected spinal sensitization in neuropathic itch. However, we cannot exclude peripheral sensitization, such as sensitization of tactile C-fibers in pruritic skin in BRP (Hashimoto and Yosipovitch, 2019). Since we found the most extensive branching in the two pruritic areas with alloknesis, increased excitability (Barkai et al., 2020; Wong et al., 2022) and an expanded receptive field by increased branching could enhance the spinal pruriceptive input inducing alloknesis (Finnerup et al., 2021).

The electrical hyperknesis testing revealed significantly higher pruritic sensations in the PLi area in the case of AD compared with PNLi, NPNL, and HCs, both at sine and half*-*sine wave stimulation, which is remarkable when considering the reduced intraepidermal nerve fiber density in particular compared to HCs. In BRP patients, reporting of itch sensations was a bit mixed. Whereas, upon half*-*sine stimulation, patients reported higher intensities in both pruritic areas (compared to NPNL and HCs), this holds upon sine stimulation only for PNLi. Furthermore, it should be noted that the intensity of both sine and half*-*sine stimulation in the PNLi in BRP was the most intense and comparable to the PLi and PNLi in AD. This could be due to the existing NGF-mediated sensitization of C-fibers, which enhances their response to electrical stimulation in BRP (Schnakenberg et al., 2021). In both BRP and AD, all three areas tested had higher maximum itch intensity ratings than the HC subjects. This again suggests an underlying hypersensitivity in the patients, which can already be observed in the healthy-appearing skin of the patients. Importantly, sensitization to electrical stimulation by extreme branching (Barkai et al., 2020; Wong et al., 2022) might primarily increase the pain sensation and thus explain the combination of higher pain ratings and lower innervation density in BRP as compared to HCs. In line with this, in patients with type 2 diabetes, immunofluorescence staining of GAP-43, a marker of neuronal regeneration, showed that they had more pain with increased branching of GAP-positive fibers (Galosi et al., 2018). Moreover, higher pain ratings could also inhibit the itch intensity induced by electrical stimulation and thereby explain lower itch ratings in BRP with a higher degree of branching and more BRP patients reporting pain sensation in all three areas compared to AD. It should be noted that the type of electrostimulation has a direct influence on the response of specific fiber types, or can cause simultaneous activation of different fiber types (Solinski and Rukwied, 2021).

In summary, our data imply that inflammatory (AD) and neuropathic (BRP) chronic itch conditions are both characterized by decreased intraepidermal nerve fiber density, caused to a lesser extent by chronic scratching, but rather via more profound mechanisms. Instead, chronic scratching increases nerve fiber branching in both entities, which is particularly evident in BRP. As a sign of peripheral sensitization, electrically induced itch and pain were found to increase in symptomatic skin in both entities. However, increased cowhage-induced itch and electrically induced itch in the healthy-appearing skin of the patients indicate central sensitization for itch.

## Data availability statement

The raw data supporting the conclusions of this article will be made available by the authors, without undue reservation.

## Ethics statement

The studies involving humans were approved by Ethics Committee (Medical Faculty of the University of Münster; No: 2017-562-f-S). The studies were conducted in accordance with the local legislation and institutional requirements. The participants provided their written informed consent to participate in this study.

## Author contributions

LR: Data curation, Formal analysis, Investigation, Methodology, Validation, Visualization, Writing—original draft, Writing—review and editing. HW: Data curation, Formal analysis, Investigation, Methodology, Validation, Visualization, Writing—original draft, Writing—review and editing. BP: Methodology, Writing—review and editing. AS: Data curation, Software, Writing—review and editing. CZ: Investigation, Methodology, Writing—review and editing. MP: Formal analysis, Investigation, Methodology, Writing—review and editing. MS: Formal analysis, Methodology, Validation, Writing—review and editing. SS: Conceptualization, Funding acquisition, Project administration, Resources, Supervision, Validation, Writing—original draft, Writing—review and editing. KA: Conceptualization, Formal analysis, Funding acquisition, Methodology, Project administration, Supervision, Validation, Visualization, Writing—original draft, Writing—review and editing.
