# Supplementary material for: Scratching increases epidermal neuronal branching and alters psychophysical testing responses in atopic dermatitis and brachioradial pruritus
Source: Front Mol Neurosci. 2023 Sep 19;16:1260345. doi: 10.3389/fnmol.2023.1260345 (PMC10546039; doi:10.3389/fnmol.2023.1260345)
Supplement: Supplementary file 1 [file Data_Sheet_1.docx]

Supplementary Material

**Scratching increases epidermal neuronal branching and alters psychophysical testing responses in atopic dermatitis and brachioradial pruritus**

Lina Renkhold^1†^, Henning Wiegmann^1†^, Bettina Pfleiderer^2^, Aysenur Süer^3^, Claudia Zeidler^1^, Manuel P. Pereira^4,5,^ Martin Schmelz^6^, Sonja Ständer^1^, Konstantin Agelopoulos^1*^

*** Correspondence:** Konstantin Agelopoulos: Konstantin.Agelopoulos@ukmuenster.de

**Supplementary Tables**

**Supplementary table 1. Inclusion and exclusion criteria.** NRS: numerical rating scale

| **Inclusion criteria** |
| --- |
| Age: ≥ 18 years |
| Patient groups: Chronic pruritus (≥ 6 weeks duration) |
| Patient groups: Diagnosis of atopic dermatitis or brachioradial pruritus |
| Patient groups: presence of pruritus at the forearms |
| Patient groups: Average itch of the previous 24h on the NRS: ≥ 3/10 |
| Atopic dermatitis: presence of lichenifed lesions, eczema and non-lesional non-pruritic skin at the arms |
| Brachioradial pruritus: presence of lichenifed lesions, pruritic non-lesional skin and non-lesional non-pruritic skin at the arms |
| Healthy controls: No pruritus (NRS: 0) |
| Skin type I-IV according to Fitzpatrick classification |
| **Exclusion criteria** |
| No understanding of the German language |
| Chronic pruritus due to another condition (e.g. systemic diseases such as diabetes) |
| Condition preventing the participation in the experimental procedures |
| Acute infection, skin lesions or tattoos at testing sites |
| Acute psychiatric condition (e.g. acute depression) |
| Fibromyalgia, complex regional pain syndrome |
| Neurological conditions (e.g. Parkinson, epilepsy) |
| Previous stroke |
| HIV |
| Pacemaker |
| Tendency to excessive scarring |
| Pregnant or lactating women |
| Allergy to substances used in the study, including local anaesthetics, latex or pflaster. |
| Alcohol or drug abuse in the previous 2 years |
| Abuse of sleeping medication in the previous 2 years |
| Intake of opioid analgesics |
| Intake of non-opioid analgesics on the day of experimental procedures |
| Use of emollients with urea, polidocanol, topical keratinolytics, exfoliatives, self-tanners, cosmetics or skin care products on the day of experimental procedures |
| Intake of sedatives or sleeping pills 1 day prior to experimental procedures |
| Use of medication that may affect the perception of itch (e.g. antihistamines, topical capsaicin, topical steroids, topical calcineurin-inhibitors) 1 week prior to experimental procedures. |
| Intake of systemic steroids 2 weeks prior to experimental procedures. |
| Therapy with systemic immunomodulatory drugs, immunosuppressive drugs (e.g. MTX), biologics, phototherapy 1 month prior to experimental procedures. |
| Contraindications for MRI examination* |
| Healthy controls: atopic disposition |

**Study participants also participated in a functional MRI examination (data not shown)*

**Supplementary table 2. Scratch sign score.**

| **Scratch sign score** | |
| --- | --- |
| **I. Morphology**  (predominant lesion) | **Score** |
| No scratch lesions | 0 points |
| Group 1: Erosions, excoriations, crusts, ulcer, scars | 1 point |
| Group 2: papules, lichenification | 2 points |
| Group 3: nodules, plaques | 3 points |
| Group 4: excoriated papules, nodules and/or plaques | 4 points |
| **II. Body surface**  (1 palm corresponds to 1% body surface) | **Score** |
| No scratch lesions | 0 points |
| <10% | 1 point |
| >10-29% | 2 points |
| 30-49% | 3 points |
| 50-79% | 4 points |
| 80-100% | 5 points |
| **Total score = Morphology score x Body surface score (0-20)** | |

**Supplementary table 3. Epidermal neuroanatomical architecture.**

| **Epidermal neuroanatomical architecture*** | | | | | |
| --- | --- | --- | --- | --- | --- |
|  | | **IENFD** |  | | **IENFD** |
| **AD**  **(n=31)** | PLi | 3.77 [2.83;7.45] | **BRP**  **(n=32)** | PLi | 4.27 [2.29;5.37] |
|  | PNLi | 5.65 [3.53;7.66] |  | PNLi | 3.91 [2.70;6.50] |
|  | NPNL | 7.25 [5.09;10.04] |  | NPNL | 6.37 [4.52;8.59] |
|  | HC (n=28) | 11.40 [8.89;14.92] |  | HC (n=27) | 10.28 [8.83;13.14] |
|  | | **Branching** |  | | **Branching** |
| **AD**  **(n=31)** | PLi | 1.5 [1.0;2.0] | **BRP**  **(n=31)** | PLi | 3.0 [2.0;3.0] |
|  | PNLi | 1.0 [1.0;2.0] |  | PNLi | 2.0 [2.0;2.0] |
|  | NPNL | 1.0 [1.0;1.0] |  | NPNL | 1.0 [1.0;1.0] |
|  | HC (n=28) | 1.0 [1.0;1.0] |  | HC (n=24) | 1.0 [1.0;1.3] |
| **median [interquartile range]* | | |  |  |  |

**Supplementary table 4. Electrically-evoked itch and pain.** Study participants received half sine and sine wave stimulation at the arms. In atopic dermatitis (AD) patients and in patients with brachioradial pruritus (BRP) assessments were performed at pruritic lichenified (PLi), pruritic non-lichenifed (PNLi) and non-pruritic non-lesional skin (NPNL). Assessments in healthy controls (HC) were done in a NPNL area at the same anatomical location as the PLi area of the matched patient. Maximal evoked itch and pain intensities were recorded for both stimulation paradigms using the numerical rating scale (NRS). Additionally, perception thresholds are shown for sine stimulation. AD: atopic dermatitis, BRP: brachioradial pruritus, HC: healthy control, IQR: interquartile range, mA: milliamps, NPNL: non-pruritic non-lesional skin; NRS: numerical rating scale; PLi: pruritic lichenifed skin; PNLi: pruritic non-lichenifed skin. PLi vs. all other assessment areas (marked in bold): Related samples: Wilcoxon Test; independent samples: Mann-Whitney-U Test. *p<0.05, **p<0.01, ***p<0.001

|  | **atopic dermatitis** | | | **AD-HC** | **brachioradial pruritus** | | | **BRP-HC** |
| --- | --- | --- | --- | --- | --- | --- | --- | --- |
|  | PLi | PNLi | NPNL | HC | PLi | PNLi | NPNL | HC |
| ***Half sine stimulation*** *(median [IQR])* | | | | | | | | |
| Itch_max_-NRS: | 4.5 [3.3;7.8] (n=16) | **3.5 [2.0;5.8]* (n=16)** | **2.5 [0.0;3.0]** (n=16)** | **0.0 [0.0;1.5]*** (n=12)** | 3.5 [1.8;7.5] (n=14) | 4.5 [1.5;7.0] (n=14) | **2.0 [0.0;3.5]* (n=14)** | **0.0 [0.0;0.0]*** (n=14)** |
| Pain_max_-NRS: | 6.5 [5.0;8.0] (n=34) | 6.5 [5.0;8.0] (n=34) | 5.0 [4.0;8.0] (n=34) | 6.0 [3.0;7.3] (n=30) | 8.0 [6.0;9.0] (n=33) | 8.0 [4.5;9.0] (n=33) | 8.0 [6.5;10.0] (n=33) | 6.0 [4.0;8.0] (n=33) |
| ***Sine stimulation*** *(median [IQR])* | | | | | | | | |
| Sine threshold (mA): | 0.05 [0.025;0.1] (n=33) | 0.0375 [0.025;0.05] (n=34) | **0.025 [0.025;0.05]* (n=34)** | 0.025 [0.025;0.05] (n=30) | 0.025 [0.025;0.05] (n=34) | 0.025 [0.025;0.05] (n=34) | 0.025 [0.021;0.05] (n=34) | 0.025 [0.018;0.05] (n=33) |
| Itch_max_-NRS: | 3.0 [1.0;7.0] (n=19) | 3.0 [1.0;6.0] (n=19) | **1.0 [0.0;3.0]** (n=19)** | **0.0 [0.0;1.5]*** (n=17)** | 3.0 [2.0;5.0] (n=18) | 4.5 [2.0;7.3] (n=18) | 1.5 [1.0;3.3] (n=18) | **0.0 [0.0;0.0]*** (n=17)** |
| Pain_max_-NRS: | 5.5 [4.0;8.0] (n=34) | 6.0 [4.0;8.0] (n=34) | 6.0 [4.0;8.0] (n=34) | 6.0 [3.0;7.3] (n=30) | 8.0 [4.5;9.0] (n=33) | **8.0 [5.0;10.0]* (n=33)** | 8.0 [6.0;9.0] (n=33) | 7.0 [4.5;8.5] (n=33) |


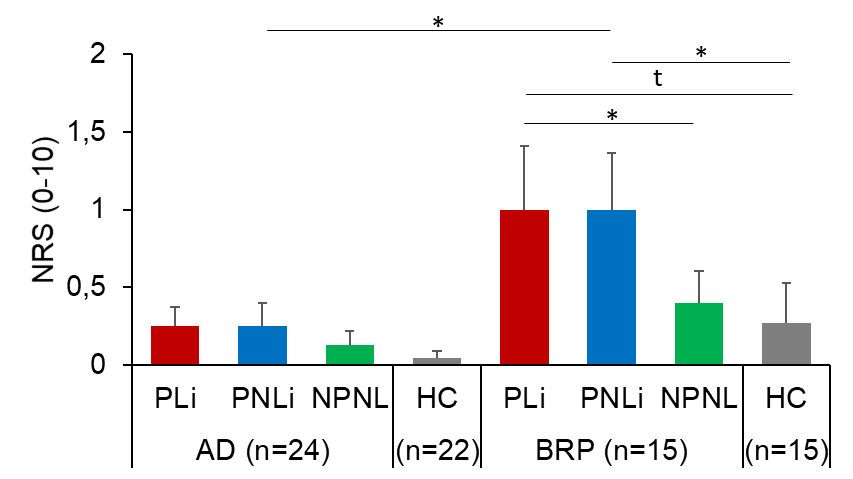


**Supplementary Figure 1.** **Alloknesis-induced maximal itch ratings of AD and BRP patients.** Bar graphs showing the maximal perceived mechanically-induced itch intensity from AD (n=24) and BRP (n=15) patients at pruritic lichenified (PLi), pruritic non-lichenified (PNLi) and non-pruritic non-lesional skin (NPNL); and in healthy skin of matchted controls (AD: n=24, BRP: n=15). AD patients showed no differences across skin areas or compared to HC. Itch intensity was significantly higher in lichenified skin compared to NPNL skin of BRP patients (p=0.038). Additionally, in PNLi skin of BRP patients itch ratings were significantly higher as in HC (p=0.04) and as in the corresponding area of AD patients (p=0.042). For statistical reasons, all patients were included in the calculation. This obviously leads to an overall low maximal NRS value. AD: atopic dermatitis; BRP: brachioradial pruritus: HC, healthy controls; NPNL: non-pruritic non-lesional skin; NRS: numerical rating scale (range 0-10); PLi: pruritic lichenified skin; PNLi: pruritic non-lichenified skin. Related samples: Wilcoxon Test; independent samples: Mann-Whitney-U Test. t: Trend p<0.1; *p<0.05.


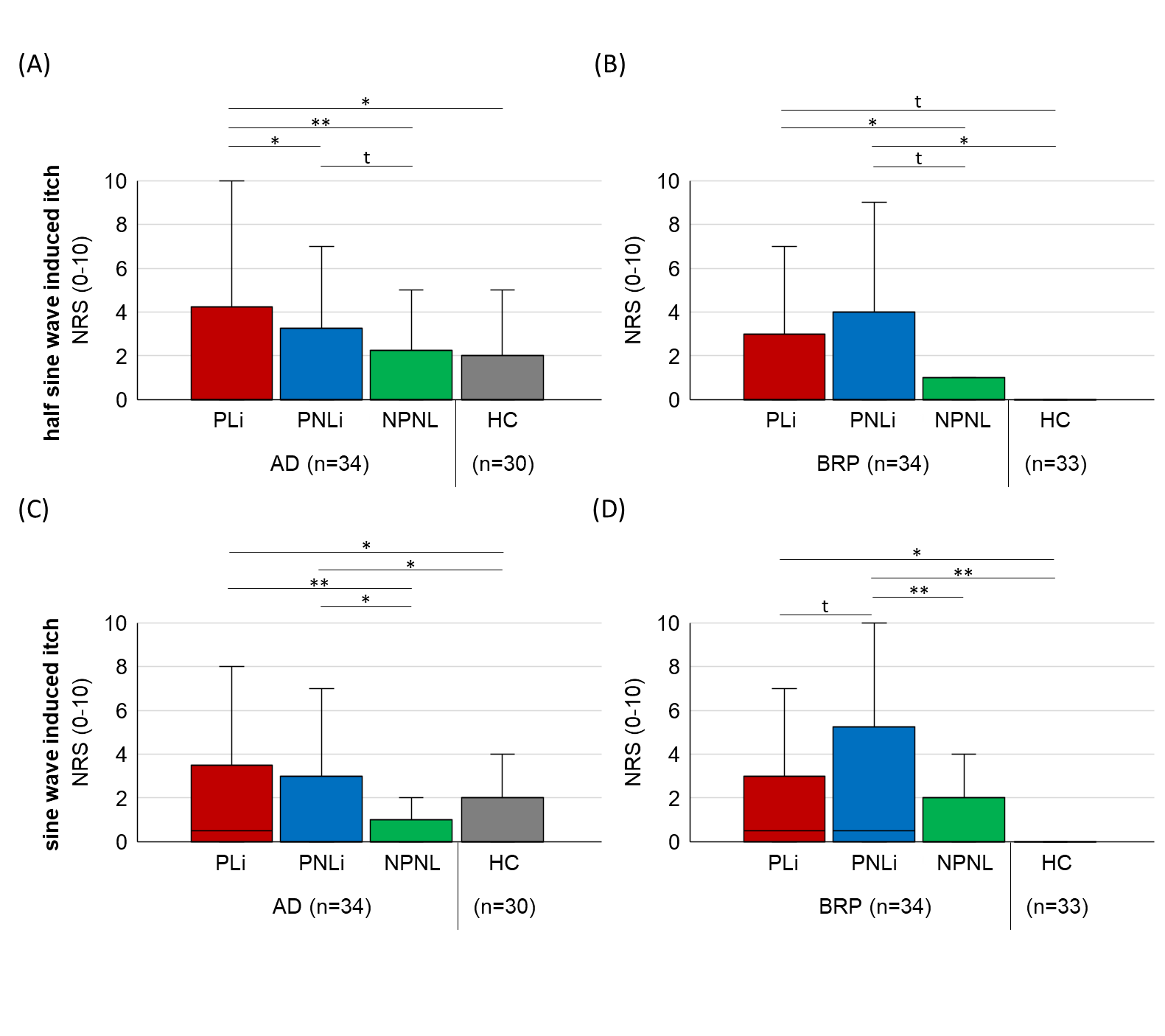


**Supplementary Figure 2. Electrically-induced itch max ratings of all AD and BRP patients. (A)**, **(B)** Boxplots of electrical half sine wave-induced maximal itch intensities (NRS) in PLi, PNLi and NPNL skin of AD (n=34) and BRP (n=34) patients as well as in NPNL skin of matched HC (n=30 and n=33, respectively). **(C)**, **(D)** Electrical sine wave-induced maximal itch intensities (NRS) in PLi, PNLi and NPNL skin of AD (n=34) and BRP (n=34) patients as well as in NPNL skin of matched HC (n=30 and n=33). **(A)** Half sine stimulation evoked increased itch in lichenified skin of AD patients compared to PNLi (p=0.016), to NPNL (p=0.003) and to controls (p<0.05). **(B)** In BRP patients, itch intensity was higher in lichenified skin compared to NPNL skin (p=0.048) and higher PNLi skin compared to HC (p<0.05). **(C)** According to sine wave stimulation of AD patients, higher NRS ratings were observed in lichenified skin as in NPNL skin (p=0.002) and in HC (p<0.05) as well as in PNLi compared to NPNL skin (p=0.032) and HC (p<0.05). **(D)** BRP-matched HC reported significantly lower itch ratings compared to BRP patients in lichenified (p<0.05) and PNLi skin (p<0.05) and patients reported increased itch intensity in PNLi skin compared to NPNL skin (p=0.002). AD: atopic dermatitis; BRP: brachioradial pruritus; HC: healthy control; NPNL: non-pruritic non-lesional skin; NRS: numerical rating scale; PLi: pruritic lichenifed skin; PNLi: pruritic non-lichenifed skin; t: trend. Related samples: Wilcoxon Test; independent samples: Mann-Whitney-U Test. t: p<0.1, *p<0.05, **p<0.01, ***p<0.001
